# Impact of COVID-19 Pandemic on Young Children With Feeding and Eating Problems and Disorders and Their Families

**DOI:** 10.1097/MPG.0000000000003563

**Published:** 2022-07-15

**Authors:** Hilde Krom, Joost van Mameren, Lianne Remijn, Katinka de Nennie, Eric Dumont, Ellen van der Gaag, Marianne C. C. van Leeuwen, Sandra Mulkens, Chantal Schakelaar, Angelika Kindermann

**Affiliations:** From the * Department of Pediatric Gastroenterology, Hepatology and Nutrition, Emma Children’s Hospital, Amsterdam UMC, University of Amsterdam, Amsterdam, The Netherlands; the † SeysCentra, Malden, The Netherlands; the ‡ Multidisciplinary Advisory Board, Patient organization “Nee-eten,” Haarlem, The Netherlands; the § Institute of Physics, University of Amsterdam, Amsterdam, The Netherlands; the ‖ HAN University of Applied Sciences, Academy of Health Studies, Nijmegen, The Netherlands; the ¶ Pediatric Dietetic Practice Katinka de Nennie, Gouda/Zoetermeer, The Netherlands; the # Department of Clinical Psychological Science, Maastricht University, Maastricht, The Netherlands; the ** Department of Psychiatry and Neuropsychology, School for Mental Health and Neuroscience, Maastricht University, Maastricht, The Netherlands; the †† Pediatric Department Hospital Group Twente, Almelo-Hengelo, The Netherlands; the ‡‡ Isa Power, Udenhout, The Netherlands.

**Keywords:** avoidant/restrictive food intake disorder, COVID-19, pediatric feeding disorder, pediatrics

## Abstract

The incidence of feeding and eating problems and disorders (FEPD) in children increased during the coronavirus disease 2019 (COVID-19) pandemic. The aim of this study was to assess the impact of the COVID-19 pandemic on young children with FEPD and their parents.

**Methods:**

Cross-sectional survey: parents of children with FEPD (0–11 years) in the Netherlands completed an online questionnaire (January–April 2021). This questionnaire included 4 demographic questions (including criteria of pediatric feeding disorder [PFD] and/or avoidant/restrictive food intake disorder [ARFID]) and 11 questions related to experienced impact of the COVID-19 pandemic. Parental responses regarding children with FEPD (including PFD and ARFID) were compared with those of healthy controls (HCs).

**Results:**

In total, 240 children (median age, 5.5 years; interquartile range [IQR], 3.5–7.9 years; 53.3% female) were included; 129 children with FEPD and 111 HC. Most children with FEPD fulfilled criteria for PFD (n = 119; 92.2%) and/or ARFID (n = 117; 90.7%). Parents of children with FEPD reported more stress (of their child [*P* = 0.014] and parental stress [*P* = 0.014]), worse eating by the child (*P* < 0.001), more negative relations within the family (*P* = 0.006), and less support from the environment (*P* = 0.001) compared with parents of HC during the COVID-19 pandemic than before.

**Conclusions:**

It seems that the COVID-19 pandemic had great impact on young children with FEPD and their parents because parents of children with FEPD reported significantly more perceived stress within both the child and parents, more difficult eating behavior of the child, more negative behavior between family members, and less support from the environment as compared with HC.

**What Is Known**

- The incidence of feeding and eating problems and disorders (FEPDs) in children increased during the coronavirus disease 2019 (COVID-19) pandemic.
- Before the pandemic, parents of children with avoidant/restrictive food intake disorder (ARFID) experienced a lack of support.

**What Is New**

- Parents of children with FEPD reported worse eating of the child, more stress within child and parent(s) and more negative familial relations during the COVID-19 pandemic (than before the pandemic) more often as compared with healthy controls.
- Parents of children with FEPD reported less support during the COVID-19 pandemic (than before the pandemic) more often as compared with parents of healthy controls.

The coronavirus disease 2019 (COVID-19) outbreak is caused by severe acute respiratory syndrome coronavirus 2 (SARS-CoV-2) (1–4). Similar to other countries, the Dutch government implemented restrictions to prevent SARS-CoV-2 transmission, such as home-based working, closure of schools/daycares, home-based schooling, restraining sportive activities, and restrictions in visits and social contacts (5). As a result, the COVID-19 pandemic and subsequent restrictions have impacted children and their families (6–8). In general, the COVID-19 pandemic negatively influenced parental mental health, psychosocial wellbeing, and socioeconomic situation (8–10). Many parents who kept their job switched to working from home, often while taking care of the children’s homeschooling at the same time (10). Concerns regarding domestic violence and child maltreatment have increased worldwide (10,11).

For children, the COVID-19 pandemic resulted in reduced peer contact and daily structure, homeschooling, and high rates of internalizing (such as boredom, anxiety, depression, posttraumatic stress symptoms) and externalizing symptoms (such as clinging, inattention, irritability, anger, and hyperactivity), which may all have developmental implications (5–13). On the other hand, COVID-19 restrictions might have led to opportunities for enhanced family cohesion due to increased presence of parents at home (10).

In accordance, the incidence of feeding and eating problems and disorders seems to have increased during the COVID-19 pandemic (14). This study aimed to investigate feeding problem-related outcomes during the COVID-19 pandemic on children (0–11 years) with feeding and eating problems and disorders and their parents in the Netherlands.

## MATERIALS AND METHODS

### Participants and Procedure

In this cross-sectional survey, parents of children aged <12 years with feeding and eating problems and disorders in the Netherlands were eligible and were asked to complete a short (10 minutes) online questionnaire using Qualtrics Survey software between January 2021 and April 2021. Parents were recruited by the health care professionals and parents of the Multidisciplinary Advisory Board of the Dutch patient association “Nee-eten” (www.nee-eten.nl) for parents of children with tube feeding and/or chronic food refusal, using advertisements in their newsletter and on several online platforms. The advertisement included a link to a website, describing the aims and procedures of the study, followed by an online informed consent and the questionnaire itself. Parents of children with feeding and eating problems and disorders were asked to recruit parents of healthy children of the same age, who were included as control group. Answers of parents of children with feeding and eating problems and disorders were compared with those of healthy controls. Parents of all included children gave informed consent.

### Measures

The content of the questionnaire aiming to assess the impact of the COVID-19 pandemic on children with feeding and eating problems and disorders was developed by the authors and discussed over several online meetings. Two parents of the target group gave feedback on the content and feasibility of the questionnaire. The questionnaire contained 15 questions: 4 questions inquiring sociodemographics (age, sex, medical diagnoses, and criteria of pediatric feeding disorder [PFD] and avoidant/restrictive food intake disorder [ARFID] [see *Definitions*]), 4 questions regarding impact of the COVID-19 pandemic on the child (related stress, response to restrictions, feeding/eating behavior, treatment of the feeding and eating problems and disorders), 7 questions regarding influence on the family (parental stress, family relations, child raising, meal sessions, support from the environment, support from health care practitioners, and accessibility health care). All but 1 question on experiences during the Corona pandemic were asked using a 5-point scale, with 1 indicating the most negative consequences and 5 indicating the most positive consequences. The question on treatment of feeding and eating problems and disorders had 6 answer options.

### Definitions

Children with feeding and eating problems and disorders may fulfill criteria for both PFD and ARFID. We used the criteria for PFD suggested by Goday et al (15) (2019) including “an impaired oral nutrient intake which is not age-appropriate, and is associated with medical, nutritional, feeding skill, and/or psychosocial dysfunction.” Furthermore, the definition of ARFID from the Diagnostic and Statistical Manual of Mental Disorders, Fifth Edition (DSM-5) (16) (2013) was used. ARFID is defined as “an eating or feeding disturbance (e.g., apparent lack of interest in eating or food, avoidance based on the sensory characteristics of food, concern about aversive consequences of eating) as manifested by persistent failure to meet appropriate nutritional and/or energy needs associated with one (or more) of the following: significant weight loss (or failure to achieve expected weight gain or faltering growth in children), significant nutritional deficiency, dependence on enteral feeding or nutritional supplements, marked interference with psychosocial functioning” (16). One of the questions to be answered by the parents contained checkboxes with all these criteria. Due to overlapping criteria, patients could fulfill criteria for both PFD and ARFID.

### Statistical Analysis

Data were retrieved from Qualtrics XM Platform and imported in SPSS 26 (IBM SPSS Statistics 26), in which the data were managed and analyzed. Normality of continuous variables was tested by the Shapiro-Wilk test and eyeballing. Non-normally distributed data were described as median and interquartile range (IQR). Binary and ordinal data were described as frequencies and percentages.

Children with feeding and eating problems and disorders, PFD and ARFID were compared with healthy peers using the Mann-Whitney *U* test for non-normally distributed data, the Fisher exact test for binary data, and the chi-square test for ordinal distributed data. Significance level was set at *P* = 0.05. Due to multiple testing results were interpreted explorative.

### Medical Ethics

The Medical Ethics Committee of the Amsterdam UMC, Amsterdam, The Netherlands, confirmed that the Medical Research Involving Human Subjects Act did not apply to our study.

## RESULTS

### Patient Characteristics

In total, parents of 240 children participated and were included in the study. The median age of the children was 5.5 years (IQR, 3.5–7.9 years) and 53.3% was female. The population comprised 129 (53.8%) children with feeding and eating problems and disorders and 111 (46.3%) healthy controls. Age (*P* = 0.102) and sex (*P* = 0.243) did not differ significantly between the children with feeding and eating problems and disorders and healthy controls (Table 1). Many children with feeding and eating problems and disorders fulfilled the criteria for PFD (n = 119; 92.2%) and/or ARFID (n = 117; 90.7%). Most comorbidities were of psychiatric/psychological, gastrointestinal, and/or genetic origin.

**TABLE 1.** Baseline characteristics

Patient characteristics	Children with feeding problems	Healthy controls	*P*
Age, y (median)	5.1 (3.3–7.7)	5.8 y (IQR, 3.7–8.5 y)	0.102
Sex, male, n (%)	65 (50.4)	47 (42.3)	0.243
PFD, n (%)	119 (92.2)		NA
ARFID, n (%)	117 (90.7)		NA
Diagnoses*, n (%)			NA
Psychiatric/psychological	55 (42.6)		
Gastrointestinal	28 (21.7)		
Genetic/syndromes	13 (10.1)		
Pulmonary	6 (4.7)		
Ear, nose, throat	6 (4.7)		
Cardial	4 (3.1)		
Metabolic	4 (3.1)		
Other	13 (10.1)		

Diagnoses from ≥1 category could be included. Children with feeding problems fulfilling the criteria by Goday et al (15) for PFD. Children with feeding problems fulfilling DSM-5 criteria (16) for ARFID. Significance level was set at *P* = 0.05. ARFID = avoidant/restrictive food intake disorder; DSM-5 = Diagnostic and Statistical Manual of Mental Disorders, Fifth Edition; IQR = interquartile range; NA = not applicable; PFD = pediatric feeding disorder.

### Questionnaire

Results from the questionnaire related to the child and the parents are shown in Tables 2 and 3, respectively. Parents of children with feeding and eating problems and disorders reported increased stress in the child during the COVID-19 pandemic significantly more often than parents of healthy children (*P* = 0.014). Furthermore, they reported changed eating behavior by the child during the COVID-19 pandemic more often as compared with healthy controls (*P* < 0.001). In addition, overall eating behavior in children with feeding and eating problems and disorders during the COVID-19 pandemic deteriorated (questionnaire options “much more difficult” and “more difficult”) but improved eating behavior (options “better” and “much better”) more often as well, whereas parents of healthy children more often reported eating to be the same as before the pandemic (Table 2).

**TABLE 2.** COVID-19 pandemic-related measures in the child

Child	n (%)	n (%)	n (%)	n (%)	n (%)	*P*
Stress during pandemic as compared with before						
	Much more	More	Same	Less	Much less	Compared with HC
Feeding problems	12 (9.3)	24 (18.6)	45 (34.9)	9 (7.0)	10 (7.8)	**0.014**
PFD	12 (10.1)	24 (20.2)	45 (37.8)	8 (6.7)	10 (8.4)	**0.014**
ARFID	12 (10.3)	24 (20.5)	44 (37.6)	7 (6.0)	10 (8.5)	**0.011**
HC	7 (6.3)	18 (16.2)	69 (62.2)	9 (8.1)	2 (1.8)	
Response to governmental restrictions (such as “being angry; sad; frustrated”)						
	Very hard	Hard	Neutral	Good	Very good	
Feeding problems	6 (4.7)	26 (20.2)	51 (39.5)	14 (10.9)	3 (2.3)	0.155
PFD	6 (5.0)	26 (21.8)	51 (42.9)	13 (10.9)	3 (2.5)	0.119
ARFID	6 (5.1)	26 (22.2)	49 (41.9)	13 (11.1)	3 (2.6)	0.116
HC	3 (2.7)	17 (15.3)	55 (49.5)	26 (23.4)	4 (3.6)	
	Much more difficult	More difficult	Same	Better	Much better	
Eating behavior during pandemic as compared with before						
Feeding problems	10 (7.8)	25 (19.4)	43 (33.3)	16 (12.4)	6 (4.7)	**<0.001**
PFD	10 (8.4)	25 (21.0)	43 (36.1)	15 (12.6)	6 (5.0)	**<0.001**
ARFID	10 (8.5)	24 (20.5)	43 (36.8)	15 (12.8)	5 (4.3)	**<0.001**
HC	1 (0.9)	6 (5.4)	86 (77.5)	10 (9.0)	2 (1.8)	
	Same mode/time	Adapted	Not at all	Postponed	NA	
Treatment/therapies during pandemic as compared with before						
Feeding problems	11 (8.5)	54 (41.9)	14 (10.9)	4 (3.1)	17 (13.2)	NA
PFD	11 (9.2)	54 43.4)	14 (11.8)	4 (3.4)	16 (13.4)	NA
ARFID	11 (9.4)	53 (45.3)	14 (12.0)	4 (3.4)	15 (12.8)	NA
HC	NA	NA	NA	NA	NA	

Total n = 129 children with feeding (of whom n = 119 fulfilled criteria PFD and n = 117 ARFID) and n = 111 HCs. Missing data in feeding problems (n = 29), PFD (n = 20), ARFID (n = 20), and HCs (n = 6) (same numbers for each question). Children with feeding problems fulfilling DSM-5 criteria (16) for ARFID. Children with feeding problems fulfilling the criteria by Goday et al (15) for PFD. Significance level was set at *P* = 0.05. Bold values indicate *P* < 0.05. ARFID = avoidant/restrictive food intake disorder; COVID-19 = coronavirus disease 2019; DSM-5 = Diagnostic and Statistical Manual of Mental Disorders, Fifth Edition; HCs = healthy controls; NA = not applicable; PFD = pediatric feeding disorder.

**TABLE 3.** COVID-19 pandemic-related measures in parents and family

Parents and family	n (%)	n (%)	n (%)	n (%)	n (%)	n (%)	*P*
	Much more	More	Same	Less	Much less		
Stress during pandemic as compared with before							
Feeding problems	17 (13.2)	46 (35.7)	13 (10.1)	7 (5.4)	3 (2.3)		**0.014**
PFD	17 (14.3)	46 (38.7)	13 (10.9)	7 (5.9)	3 (2.5)		**0.014**
ARFID	17 (14.5)	45 (38.5)	13 (11.1)	7 (6.0)	3 (2.6)		**0.015**
HC	7 (6.3)	41 (36.9)	32 (28.8)	9 (8.1)	3 (2.7)		
	Much more negative	More negative	Same	More positive	Much more positive		
Behavior between family members							
Feeding problems	4 (3.1)	30 (23.3)	28 (21.7)	18 (14.0)	6 (4.7)		**0.006**
PFD	4 (3.4)	30 (25.2)	28 (23.5)	18 (15.1)	6 (5.0)		**0.006**
ARFID	4 (3.4)	30 (25.6)	28 (23.9)	17 (14.5)	6 (5.1)		**0.006**
HC	3 (2.7)	16 (14.4)	50 (45.0)	22 (19.8)	1 (0.9)		
	Much more difficult	More difficult	Same	Easier	Much easier		
Child raising							
Feeding problems	7 (5.4)	47 (36.4)	26 (20.2)	5 (3.9)	1 (0.8)		0.114
PFD	7 (5.9)	47 (39.5)	26 (21.8)	5 (4.2)	1 (0.8)		0.114
ARFID	7 (6.0)	47 (40.2)	25 (21.4)	5 (4.3)	1 (0.9)		0.091
HC	3 (2.7)	38 (34.2)	45 (40.5)	5 (4.5)	1 (0.9)		
	Much harder	Harder	Same	Less hard	Much less hard		
Meal sessions							
Feeding problems	8 (6.2)	33 (25.6)	28 (21.7)	15 (11.6)	2 (1.6)		**<0.001**
PFD	8 (6.7)	33 (27.7)	28 (23.5)	15 (12.6)	2 (1.7)		**<0.001**
ARFID	8 (6.8)	33 (28.2)	28 (23.9)	15 (12.8)	1 (0.9)		**<0.001**
HC	0 (0.0)	8 (7.2)	71 (64.0)	11 (9.9)	2 (1.8)		
	Much less	Less	Same	More	Much more		
Support from environment							
Feeding problems	11 (8.5)	31 (24.0)	39 (30.2)	3 (2.3)	2 (1.6)		**0.001**
PFD	11 (9.2)	31 (26.1)	39 (32.8)	3 (2.5)	2 (1.7)		**0.001**
ARFID	11 (9.4)	31 (26.5)	38 (32.5)	3 (2.6)	2 (1.7)		**0.001**
HC	1 (0.9)	22 (19.8)	60 (54.1)	9 (8.1)	0 (0.0)		
	Much less	Less	Same	More	Much more	NA	
Support from health care professionals							
Feeding problems	15 (11.6)	24 (18.6)	37 (28.7)	2 (1.6)	2 (1.6)	6 (4.7)	NA
PFD	15 (12.6)	24 (20.2)	37 (31.1)	2 (1.7)	2 (1.7)	6 (5.0)	NA
ARFID	15 (12.8)	24 (20.5)	36 (30.8)	2 (1.7)	2 (1.7)	6 (5.1)	NA
HC	NA	NA	NA	NA	NA		
	Much worse	Worse	Same	Better	Much better	NA	
Accessibility health care							
Feeding problems	11 (8.5)	29 (22.5)	41 (31.8)	1 (0.8)	0 (0.0)	4 (3.1)	NA
PFD	11 (9.2)	29 (24.4)	41 (34.5)	1 (0.8)	0 (0.0)	4 (3.4)	NA
ARFID	11 (9.4)	29 (24.8)	40 (34.2)	1 (0.9)	0 (0.0)	4 (3.4)	NA
HC	NA	NA	NA	NA	NA		

Total n = 129 children with feeding (of whom n = 119 fulfilled criteria PFD and n = 117 ARFID) and n = 111 HCs. Missing data in feeding problems (n = 43), PFD (n = 33), ARFID (n = 32), and HCs (n = 19) (same numbers for each question). Children with feeding problems fulfilling DSM-5 criteria (16) for ARFID. Children with feeding problems fulfilling the criteria by Goday et al (15) for PFD. Significance level was set at *P* = 0.05. Bold values indicate *P* < 0.05. ARFID = avoidant/restrictive food intake disorder; COVID-19 = coronavirus disease 2019; DSM-5 = Diagnostic and Statistical Manual of Mental Disorders, Fifth Edition; HCs = healthy controls; NA = not applicable; PFD = pediatric feeding disorder.

During the pandemic, parents reported increased parental stress significantly more often compared with parents of healthy controls (*P* = 0.014). Furthermore, they reported more negative relationships between family members (*P* = 0.006) and less support from the environment (*P* = 0.001) during the COVID-19 pandemic significantly more often as compared with healthy controls. In addition, meal sessions were more frequently reported to be more difficult (options “more difficult” and “much more difficult”) and less difficult (options “easier” and “much easier”) compared with healthy controls (*P* < 0.001) (Table 3).

Within the group of children with feeding and eating problems and disorders, parent-reported answers on the questionnaire were not significantly different between males and females (stress of the child [*P* = 0.242], response to governmental restrictions [*P* = 0.959], eating behavior [*P* = 0.343], treatment/therapies [*P* = 0.746], parental stress [*P* = 0.142], behavior between family members [*P* = 0.481], child raising [*P* = 0.383], meal sessions [*P* = 0.975], support from environment [*P* = 0.404], support from health care practitioners [*P* = 0.739], and accessibility health care practitioners [*P* = 0.115]; results not shown).

**FIGURE 1.**
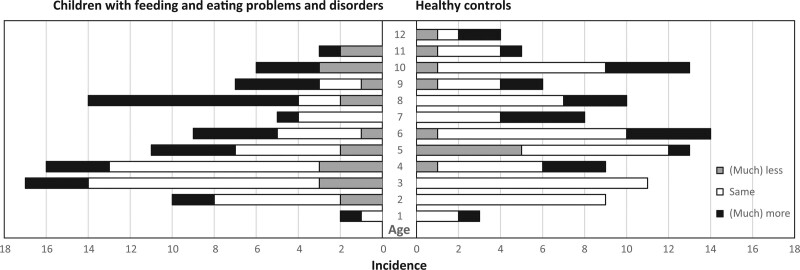
Stress within the child. Missing data due to incomplete surveys: children with FEPDs n = 29 and HCs n = 6; ages (y; mo) were rounded off to integer values. FEPDs = feeding and eating problems and disorders; HC = healthy control.

Dividing the children with feeding and eating problems and disorders in young (0 < 4 years of age) and older (4 < 12 years of age), significant differences were found on the parent-reported answers regarding stress of the child (*P* = 0.002) and accessibility of health care practitioners (*P* < 0.001). Parents of children between 0 and 4 years reported the answer “same stress” more often than parents of children between 4 and 12 years (Fig. 1). Accessibility of health care practitioners was “worse” or “much worse” in 22 of 36 children aged 0 < 4 years (61%), and in 18 of 50 children between 4 < 12 years of age (36%).

Parent-reported answers on the other questions were not significantly different (response to governmental restrictions [*P* = 0.319], eating behavior [*P* = 0.423], treatment/therapies [*P* = 0.085], parental stress [*P* = 0.507], behavior between family members [*P* = 0.733], child raising [*P* = 0.276], meal sessions [*P* = 0.847], support from environment [*P* = 0.724], and support from health care practitioners [*P* = 0.778]; results not shown).

## DISCUSSION

Parents of children with feeding and eating problems and disorders, including those with PFD and ARFID, reported significantly more perceived stress within child and parents, more difficult eating behavior of the child and more difficult meal sessions, more negative behavior between family members, and less support from the environment during the COVID-19 pandemic as compared with healthy controls.

To the best of our knowledge, this is the first study concerning the impact of the COVID-19 pandemic on young children with feeding and eating problems and disorders and their families. Children with preexisting chronic diseases, neurodevelopmental disorders (such as autism spectrum disorder or attention deficit disorder), mental disorders, disabilities, and otherwise special needs were disproportionally affected by the COVID-19 pandemic, with a higher incidence of behavioral and psychosocial problems, parental stress, and risk of child maltreatment (6,9–11,13,17). Children with feeding and eating problems and disorders are vulnerable children (often with medical comorbidities requiring additional needs). Their parents more often reported increased stress during the COVID-19 pandemic. We hypothesize this might be explained by school/daycare closures, postponed/canceled feeding therapies, and less support from the environment during the COVID-19 pandemic. This is worrying because they already reported less support from the environment as compared with parents of healthy controls before the pandemic (18). Long-lasting restrictions such as social distancing might have played an additional role. In addition, due to home-based working and closure of daycare/schools, people in their environment might have been preoccupied with handling their own problems.

Parent-reported eating behavior of most children with feeding and eating problems and disorders and experience of family meal sessions changed; parents reported both deteriorations and improvements. We hypothesize that stress of the child and parents, postponed/canceled feeding therapies, and less support from the environment negatively impacted eating behavior and meal sessions. On the other hand, spending more time with the family (due to home-based working and daycare/school closure, less social activities, etcetera) might explain why eating behavior and meal sessions improved in other families.

In the general population, food intake and type of food might have changed during the COVID-19 pandemic. For example, a large study, including 584 parents in the United States, found that parental worries regarding their child being overweight increased. Furthermore, parental feeding practices such as more controlling and pressuring feeding behaviors increased (19). These feeding practices are associated with feeding problems and disorders in children (20) and, therefore, might be an explanation for deterioration of eating behavior during the COVID-19 period.

As we expected, stress within the child seemed to be more impacted in older (4 < 12) children by the COVID-19 pandemic (increased and decreased as well), whereas “same stress” is more often reported by parents of younger (0 < 4) children. We hypothesize that this might be explained by less changes in their daily life in this younger age-group during the COVID-19 pandemic. They were not affected by school closure, less often participate in activities outside of the family house and did not yet understand the worldwide consequences of the pandemic. Another study, however, showed that children aged 3–6 years showed psychological distress more often compared with older children (7).

In accordance with other population studies, many parents of children with feeding and eating problems and disorders in our study reported that the treatment of their child was organized differently, postponed, or canceled (10,11,17). Although effects remain uncertain, we hypothesize that this might have resulted in increased stress and deterioration of problems in some of the patients. Furthermore, feeding problems may last longer in case of postponed and canceled treatments. Both inpatient and outpatient settings for patients with feeding and eating disorders transitioned to telehealth and videoconferences in order to facilitate assessments, interdisciplinary meetings, family meetings, and individual therapeutic sessions. Some unexpected benefits were revealed such as better planning of meetings, the participation of remote persons (family members and supervisors) who otherwise might not have participated, and enhanced autonomy of the patients (21,22). Unexpected benefits of telehealth were also found in children with feeding and eating disorders. Examples included observations of family mealtimes in the home environment in which the problems occur, improved participation of parents, access to health care from remote locations, and better communication and coordination between health care professionals and children and their caregivers (14,23). A study including young children with ARFID passing an intensive daycare treatment found similar results for inpatient and telehealth behavior-analytic services as follow-up (14). Equivalent results were found in a pre-COVID-19 study assessing success rates of net-coaching versus onsite treatment in order to wean tube dependent children and gaining oral feeds as well (24).

### Limitations

Some limitations have to be taken into account. One of the limitations was the use of an unvalidated questionnaire. Another limitation was that medical diagnoses and criteria for PFD (15) and ARFID (16) were reported by parents and might have been interpreted differently by health care practitioners. Validated questionnaires to assess PFD and ARFID did not yet exist. Furthermore, patients were recruited passively and parents of children with feeding problems recruited the healthy controls, which may have led to selection bias. Furthermore, due to the cross-sectional design of the study, reports on COVID-19 pandemic impact was on one time-point during the COVID-19 pandemic (instead of repeated measurements before and during the COVID-19 pandemic), which might have led to reporting bias. Due to incomplete surveys, some data for the various survey questions were missing.

## CONCLUSIONS

This explorative study showed that parents of children with feeding and eating problems and disorders reported significantly more perceived stress within both the child and parents, more difficult eating behavior of the child, more negative behavior between family members, and less support from the environment as compared with healthy controls during the COVID-19 pandemic, suggesting that the pandemic may have had great impact on them. Since the COVID 19 pandemic is still ongoing, it is important to take both the short- and long-term impact of the governmental restrictions and delayed or differently organized treatments on children with feeding and eating problems and disorders into consideration if new policies have to be made. In order to assess the impact, future studies should assess growth and development of these children on long term and compare these outcomes with data from medical charts before the pandemic.
